# SLC25A11 serves as a novel prognostic biomarker in liver cancer

**DOI:** 10.1038/s41598-020-66837-6

**Published:** 2020-06-18

**Authors:** Guoqiang Pan, Ruobing Wang, Shengnan Jia, Yanqing Li, Yan Jiao, Nan Liu

**Affiliations:** 1 Department of Hepatobiliary and Pancreatic Surgery, The First Hospital of Jilin University, Changchun, Jilin, 130021 China; 2 Department of Anesthesiology, The First Hospital of Jilin University, Changchun, Jilin, 130021 China; 3 Department of Hepatopancreabiliary Medicine, the Second Hospital of Jilin University, Changchun, Jilin, 130041 China; 4 Department of Gastrointestinal Surgery, the Second Hospital of Jilin University, Changchun, Jilin, 130041 China; 5 Department of Pathophysiology, College of Basic Medical Sciences, Jilin University, Changchun, Jilin, 130021 People’s Republic of China

**Keywords:** Prognostic markers, Risk factors

## Abstract

Liver cancer is a disease with high mortality; it is often diagnosed at intermediate and advanced stages and has a high recurrence rate. ROS restriction and adequate energy supply play significant roles in liver cancer. SLC25A11, a member of the malate-aspartate shuttle (MAS), regulates electroneutral exchange between 2-oxoglutarate and other dicarboxylates. It transports glutathione (GSH) from the cytoplasm into mitochondria to maintain GSH levels to limit ROS production. Moreover, SLC25A11 is essential for ATP generation in cancers as it regulates NADH transportation from the cytoplasm to mitochondria. The purpose of this research was to investigate the prognostic value of SLC25A11 in liver cancer. The Cancer Genome Atlas database was used to analyze the levels of SLC25A11 in liver cancer. Fisher’s exact and chi-square tests were used to evaluate the relationship between SLC25A11 expression and clinical characteristics. Finally, we explored the value of SLC25A11 in prognosis by Cox analysis and Kaplan-Meier curves. Our results revealed that SLC25A11 was downregulated in liver cancer compared to normal controls. Low expression of SLC25A11 was associated with clinical stage, vital status, histologic grade, overall survival (OS) and relapse-free survival (RFS). Liver cancer patients with low SLC25A11 expression had shorter OS and RFS than patients with high SLC25A11 expression. Multivariate analysis showed that the expression of SLC25A11 was an independent predictor of RFS and OS. In conclusion, this study identified that SLC25A11 serves as a new prognostic marker for liver cancer.

## Introduction

Liver cancer is a key cause of cancer-related mortality, accounting for 8.2% of total cancer deaths^1^. Most liver cancer patients are diagnosed at intermediate and late stages and cannot receive curative therapies, which contributes to the low survival rates and high recurrence rates of patients. With surveillance of identifiable high-risk patients and surgical intervention for early-stage patients, the 5-year survival rate has increased to 26%^2^. However, the recurrence rate of HCC is still over 70% at 5 years even after resection^3^. Therefore, it is essential to find an early screening strategy for diagnosis and prognosis prediction in liver cancer. Unfortunately, current screening markers are unsatisfactory due to their poor sensitivity and specificity. Reliable biomarkers for to improve diagnosis and to predict prognosis after treatment are urgently required.

SLC25A11 is also known as oxoglutarate carrier (OGC), which regulates electroneutral exchange between 2-oxoglutarate and some dicarboxylates^4^. It transports glutathione (GSH) from the cytoplasm to the mitochondrial matrix together with a dicarboxylate carrier (DIC; SLC25A10)^5^. Because of the importance of the GSH system in mitochondria, the regulation of the mitochondrial glutathione (mtGSH) pool can influence the metabolic disorders caused by mitochondrial dysfunction, such as diabetic nephropathy, aging and cancer^6,7^. Moreover, as a member of the malate-aspartate shuttle (MAS) family, SLC25A11 is essential for ATP generation by NADH transportation from the cytoplasm to mitochondria^8^. Therefore, these studies suggest that SLC25A11 is a key factor in the transportation of NADH and GSH from the cytoplasm into mitochondria in cancer.

Recent studies suggest that SLC25A11 plays a role in the formation of non-small-cell lung cancer (NSCLC)^8^. In addition, it promotes liver cancer by maintaining mtGSH under cholesterol loading^9^. However, the prognostic significance of SLC25A11 expression in liver cancer has not yet been determined. The diagnostic value of SLC25A11 has been evaluated by exploring the correlations between SLC25A11 expression and clinical features from the TCGA. Our results demonstrated that SLC25A11 could be a reliable marker for diagnosis and a predictor of prognosis in liver cancer.

## Result

### Characteristics of patients in this study

Gene expression data and the clinical data were collected from the TCGA and organized. Clinical characteristics included age, sex, TNM classification, residual tumor status, radiation therapy, overall survival and relapse-free survival (Table 1).

**Table 1:** Relationship between the clinical features of liver cancer and SLC25A11 expression.

Parameter	Variables	N	SLC25A11 expression				χ^2^	P value
			High	%	Low	%		
Age	<55	117	54	27.84	63	35.39	2.122	0.145
	>=55	255	140	72.16	115	64.61		
Sex	Female	121	64	32.82	57	32.02	0.003	0.957
	Male	252	131	67.18	121	67.98		
Histological type	Fibrolamellar carcinoma	3	3	1.54	0	0.00	2.840	0.343
	Hepatocellular carcinoma	363	188	96.41	175	98.31		
	Hepatocholangiocarcinoma (mixed)	7	4	2.05	3	1.69		
Histologic grade	G1	55	35	18.23	20	11.36	9.400	0.023
	G2	178	100	52.08	78	44.32		
	G3	123	52	27.08	71	40.34		
	G4	12	5	2.60	7	3.98		
Stage	I	172	100	55.25	72	42.86	8.161	0.039
	II	87	41	22.65	46	27.38		
	III	85	36	19.89	49	29.17		
	IV	5	4	2.21	1	0.60		
T classification	T1	182	107	55.44	75	42.13	7.691	0.081
	T2	95	44	22.80	51	28.65		
	T3	80	37	19.17	43	24.16		
	T4	13	5	2.59	8	4.49		
	TX	1	0	0.00	1	0.56		
N classification	N0	253	130	66.67	123	69.49	1.797	0.404
	N1	4	1	0.51	3	1.69		
	NX	115	64	32.82	51	28.81		
M classification	M0	267	138	70.77	129	72.47	0.883	0.756
	M1	4	3	1.54	1	0.56		
	MX	102	54	27.69	48	26.97		
Radiation therapy	NO	340	177	97.25	163	98.19	0.051	0.821
	YES	8	5	2.75	3	1.81		
Residual tumor	R0	326	174	91.58	152	86.36	4.121	0.210
	R1	17	7	3.68	10	5.68		
	R2	1	1	0.53	0	0.00		
	RX	22	8	4.21	14	7.95		
Vital status	Deceased	130	56	28.72	74	41.57	6.218	0.013
	Living	243	139	71.28	104	58.43		

### Low SLC25A11 expression in liver cancer

**Figure 1:**
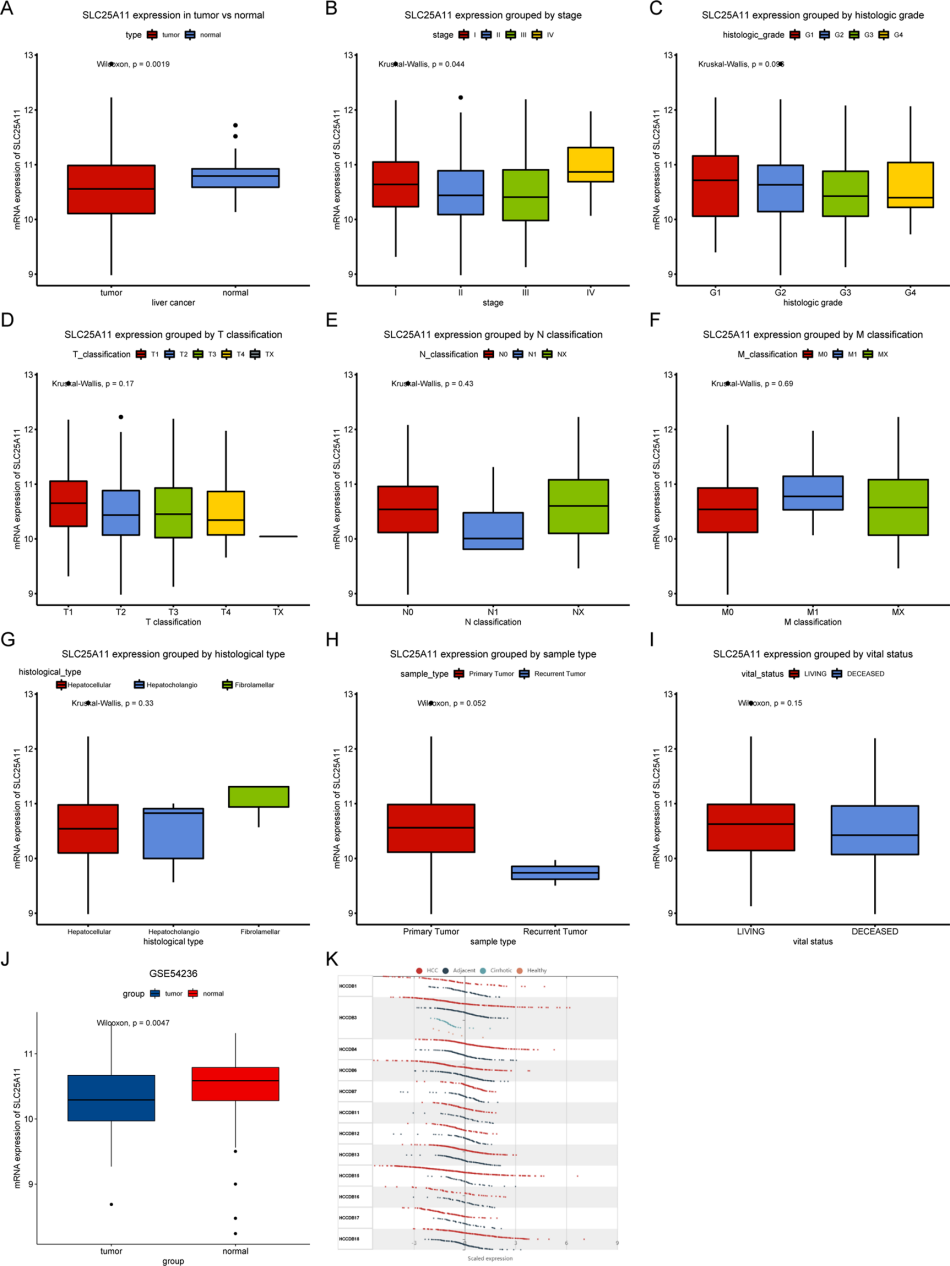
SLC25A11 expression in liver cancer. SLC25A11 expression in liver cancer tissues was compared with that in normal tissues. Subgroup analysis of SLC25A11 according to stage, histologic grade, TNM classification, histological type, sample type, and vital status. Verification in the GSE54236 and HCCDB databases.

The analysis of SLC25A11 was conducted to compare the difference in SLC25A11 expression between primary HCC tissues and normal liver tissue via box plots (Fig. 1). The results showed that SLC25A11 mRNA was downregulated in liver cancer (P = 0.0019), which was also verified by the GSE54236 and HCCDB databases (Fig. 1). In addition, we compared SLC25A11 mRNA expression among different clinical characteristics, including stage, histologic grade, TNM classification, histological type, sample type and vital status, and found that SLC25A11 mRNA expression was significantly different when patients were grouped by stage (P = 0.044) and histologic grade (P = 0.096).

### The diagnostic value of SLC25A11

**Figure 2:**
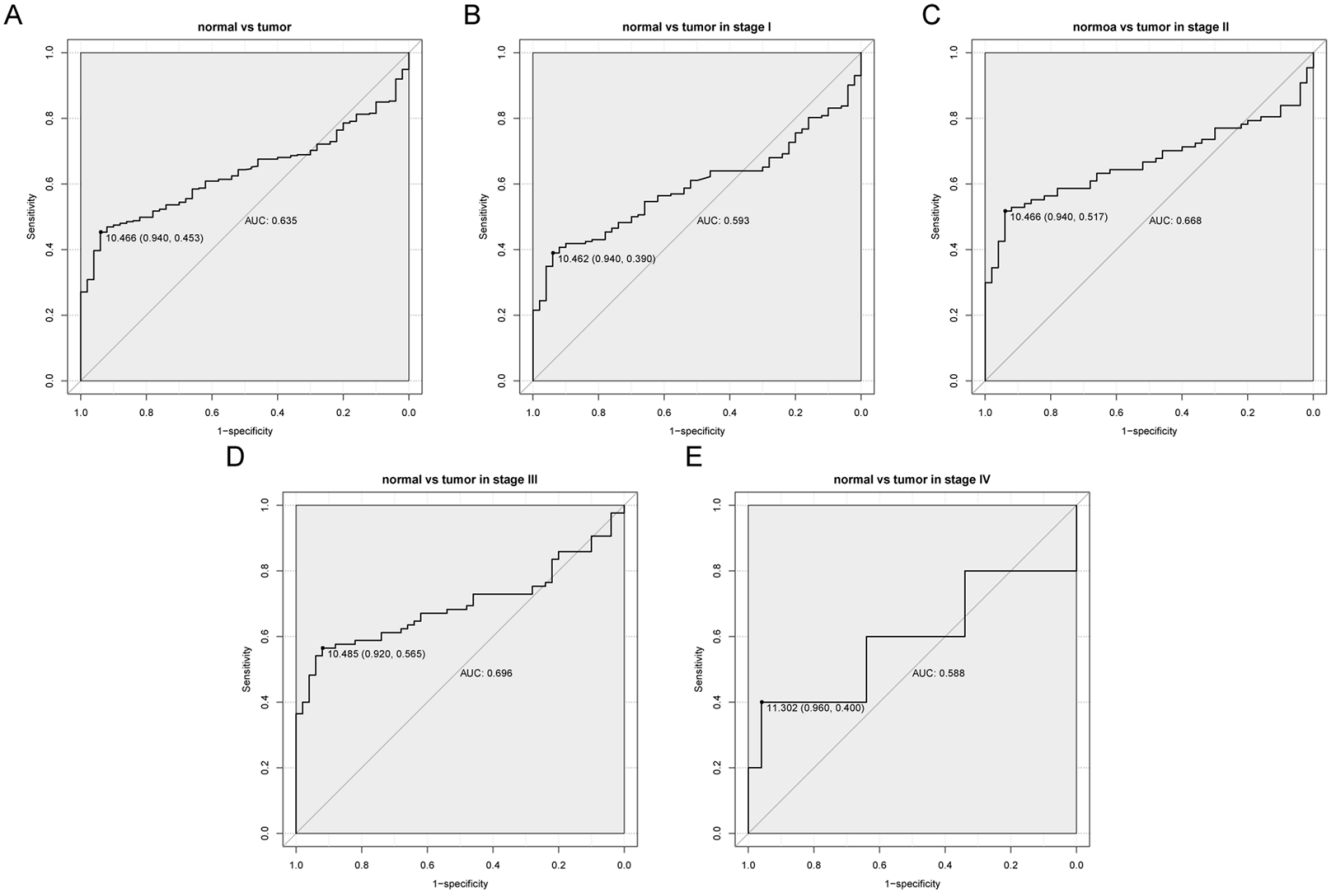
The diagnostic value of SLC25A11 mRNA expression in liver cancer. (**A**) The ROC curve of SLC25A11 expression in liver cancer tissues was compared with that of normal tissues. (**B–E**) Subgroup analysis for stage I, II, III, and IV liver cancer.

The diagnostic value of SLC25A11 was evaluated by ROC curve analysis using data from the TCGA. The area below the ROC curve (AUC) was 0.635, suggesting the modest diagnostic value of SLC25A11 (Fig. 2). Subgroup analysis revealed the diagnostic value in different stages of liver cancer with AUC values of 0.593, 0.668, 0.696, and 0.588 for stage I, stage II, stage III, and stage IV, respectively (Fig. 2).

### Correlations between SLC25A11 and clinical features with liver cancer

After dividing the patients into low/high SLC25A11 mRNA groups according to the threshold value determined by the ROC curve, the relationships between clinical features and SLC25A11 expression were analyzed (Table 1). Histologic grade (P = 0.0244), clinical stage (P = 0.0428) and vital status (P = 0.0126) were significantly correlated with SLC25A11 expression.

### Low SLC25A11 predicts poor prognosis of OS and RFS

**Figure 3:**
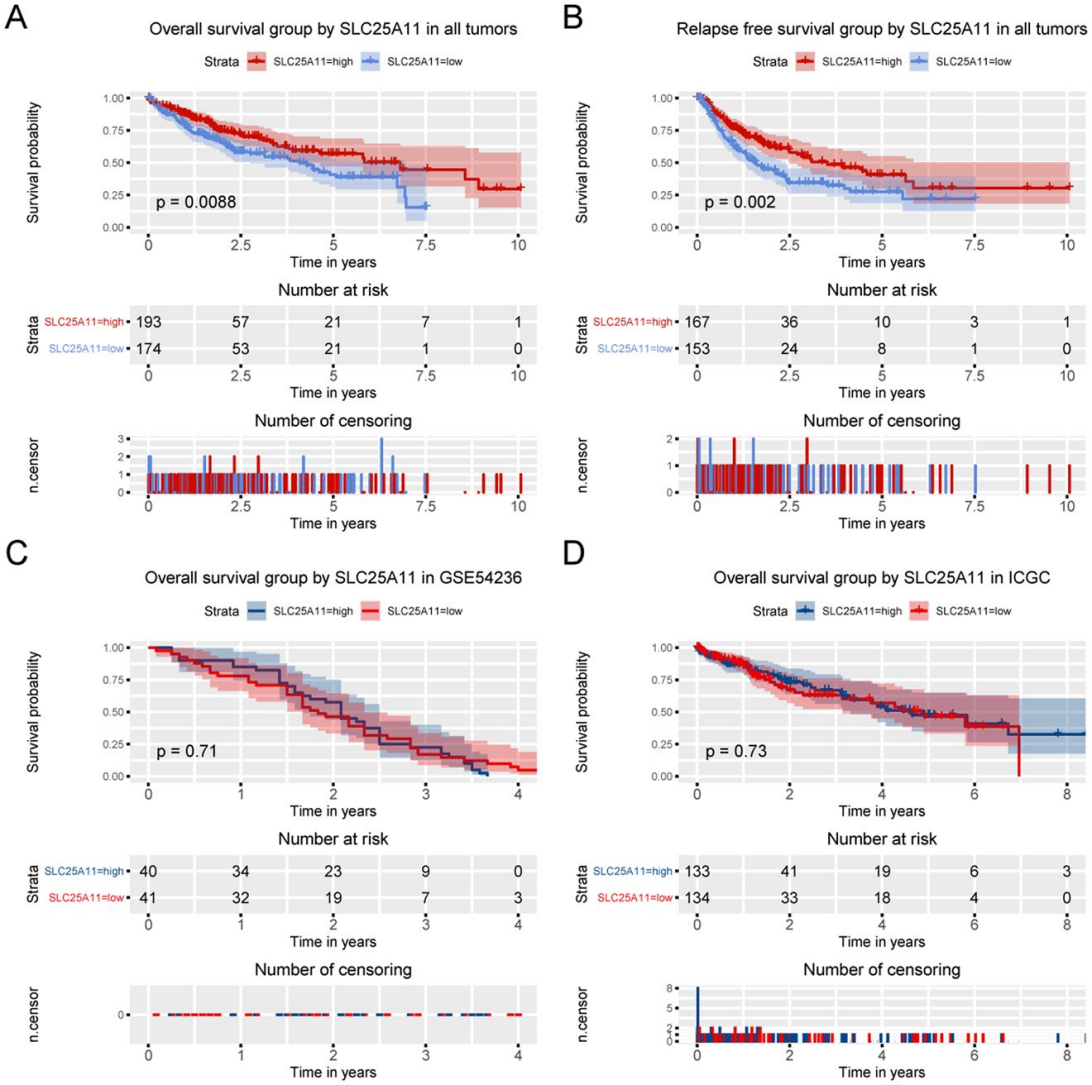
Kaplan-Meier curves for OS and RFS according to SLC25A11 expression in liver cancer. Survival analysis of OS and RFS was performed based on Kaplan-Meier curves in TCGA database, GSE54236 and HCCDB database.

Kaplan-Meier survival curves were constructed to explore the prognostic value of SLC25A11 in OS. The results showed that low expression of SLC25A11 was related to worse OS (P = 0.0088; Fig. 3). In addition, a survival curve was constructed to test the prognostic value of SLC25A11 in RFS. The results showed that low SLC25A11 expression was associated with poorer RFS than high SLC25A11 expression (P = 0.002; Fig. 3), which was also confirmed by using the ICGC and GSE54236 databases (Fig. 3).

### Subgroup analysis revealed the prognostic value of SLC25A11 in terms of OS

**Figure 4:**
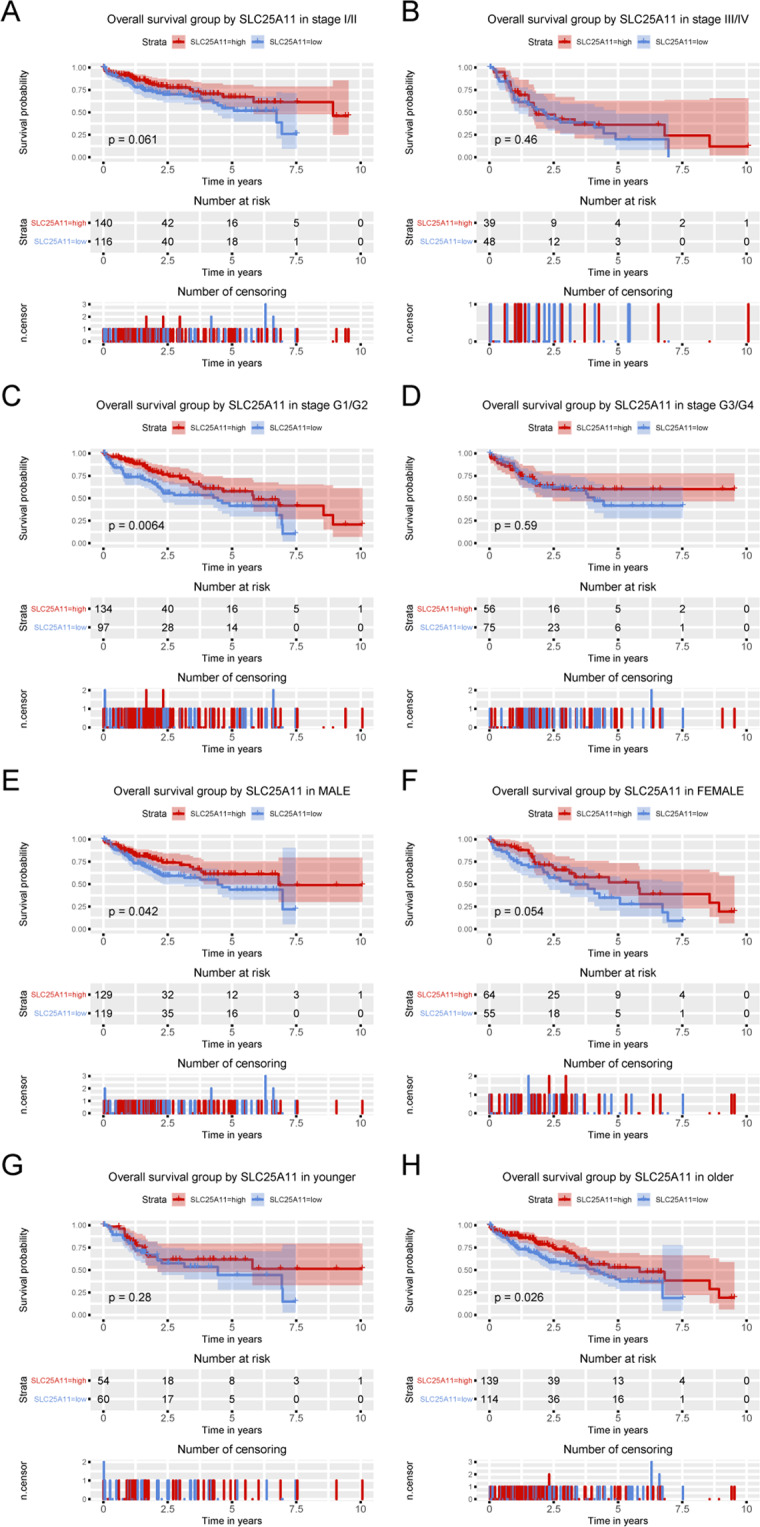
Kaplan-Meier curves for OS according to SLC25A11 expression in liver cancer. Subgroup analysis of OS was performed based on Kaplan-Meier curves according to clinical stage, histological grade, sex and age.

Subgroup analysis revealed that the low expression of SLC25A11 was correlated with poor OS in grade G1 and G2 patients (P = 0.0061), older patients (P = 0.026), and male patients (P = 0.042) (Fig. 4).

### Subgroup analysis revealed the prognostic value of SLC25A11 in terms of RFS

**Figure 5:**
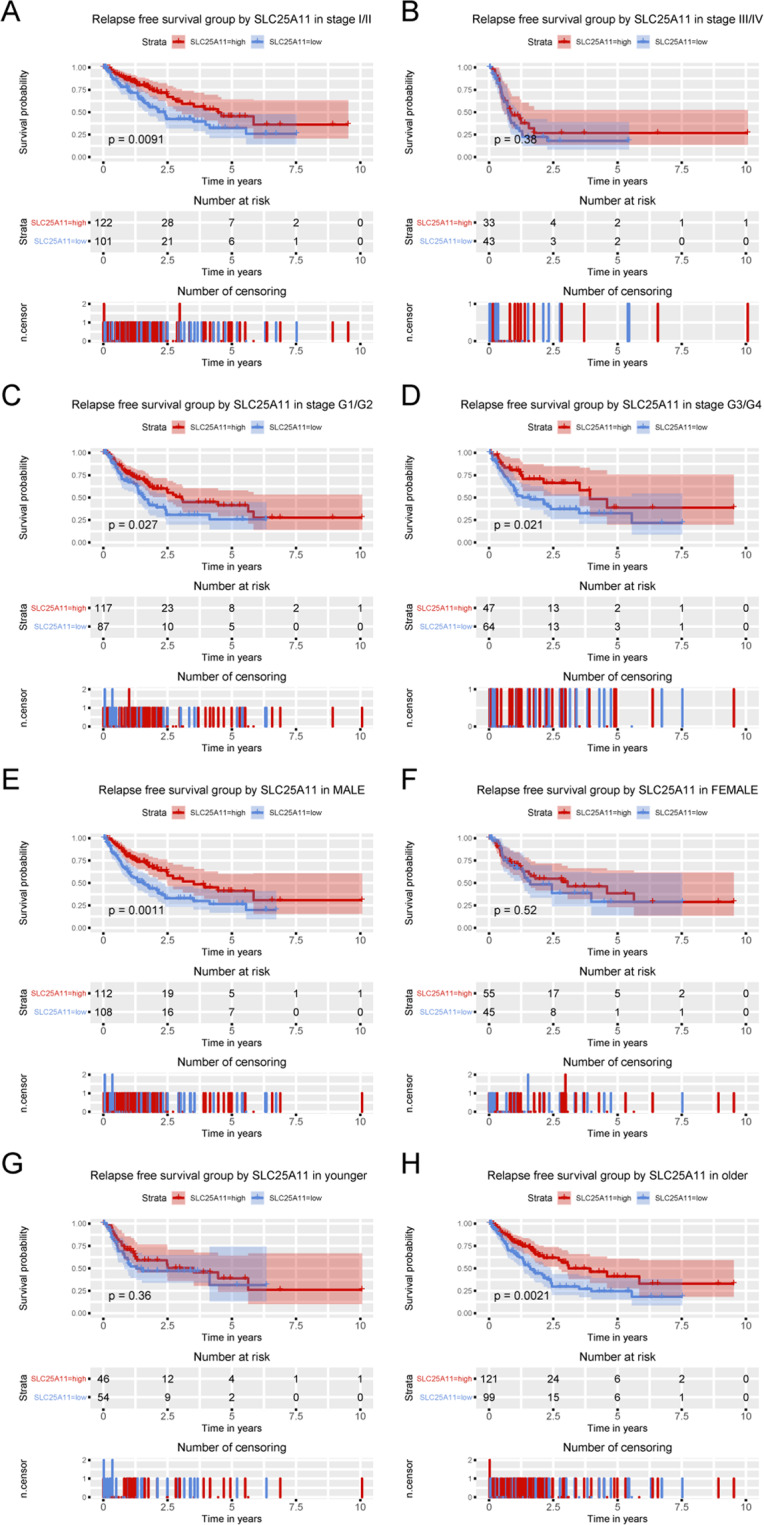
Kaplan-Meier curves for RFS according to SLC25A11 expression in liver cancer. Subgroup analysis of RFS was performed based on Kaplan-Meier curves according to clinical stage, histological grade, sex and age.

Subgroup analysis revealed that low expression of SLC25A11 was correlated with poor OS in stage I and II patients (P = 0.0091), G1 and G2 patients (P = 0.027), G3 and G4 patients (P = 0.021), male patients (P = 0.0011) and older patients (P = 0.0021) (Fig. 5).

### Low SLC25A11 is an independent predictor of OS

**Figure 6:**
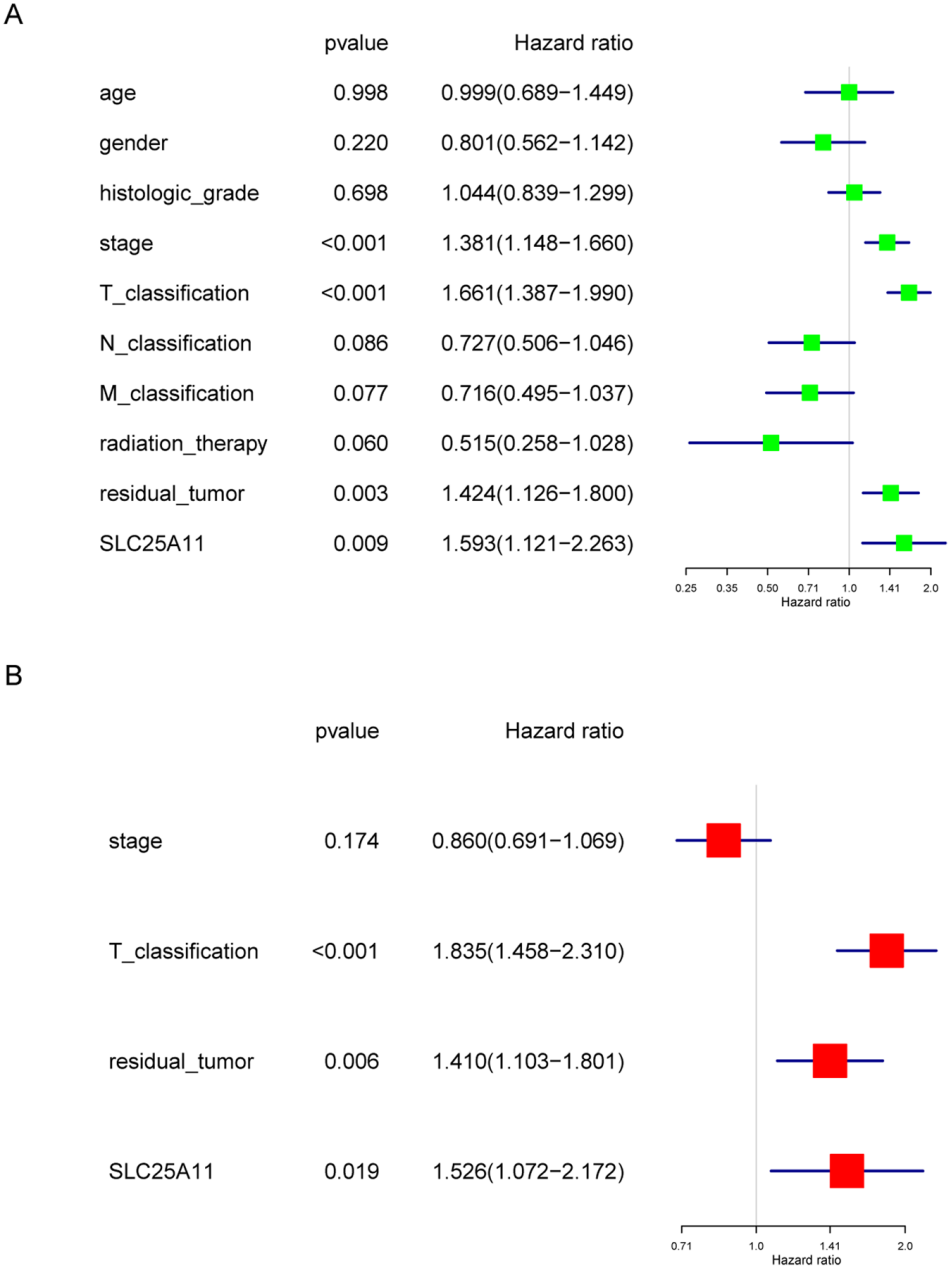
Cox model of OS among liver cancer patients. Univariate and multivariate analysis of the correlation of SLC25A11 expression with OS among liver cancer patients.

Univariate Cox analysis identified the potential OS-related variables, including the stage, T classification, residual tumor status and SLC25A11. Multivariate Cox analyses showed that SLC25A11 expression (HR value: 1.526, 95% CI: 1.072–2.172, P = 0.019), residual tumor status (HR value: 1.410, 95% CI: 1.103–1.801, P = 0.006), and T classification (HR value: 1.835, 95% CI: 1.458–2.310, P < 0.001) were independent predictors of poor OS (Fig. 6).

### Low SLC25A11 is an independent predictor of RFS

**Figure 7:**
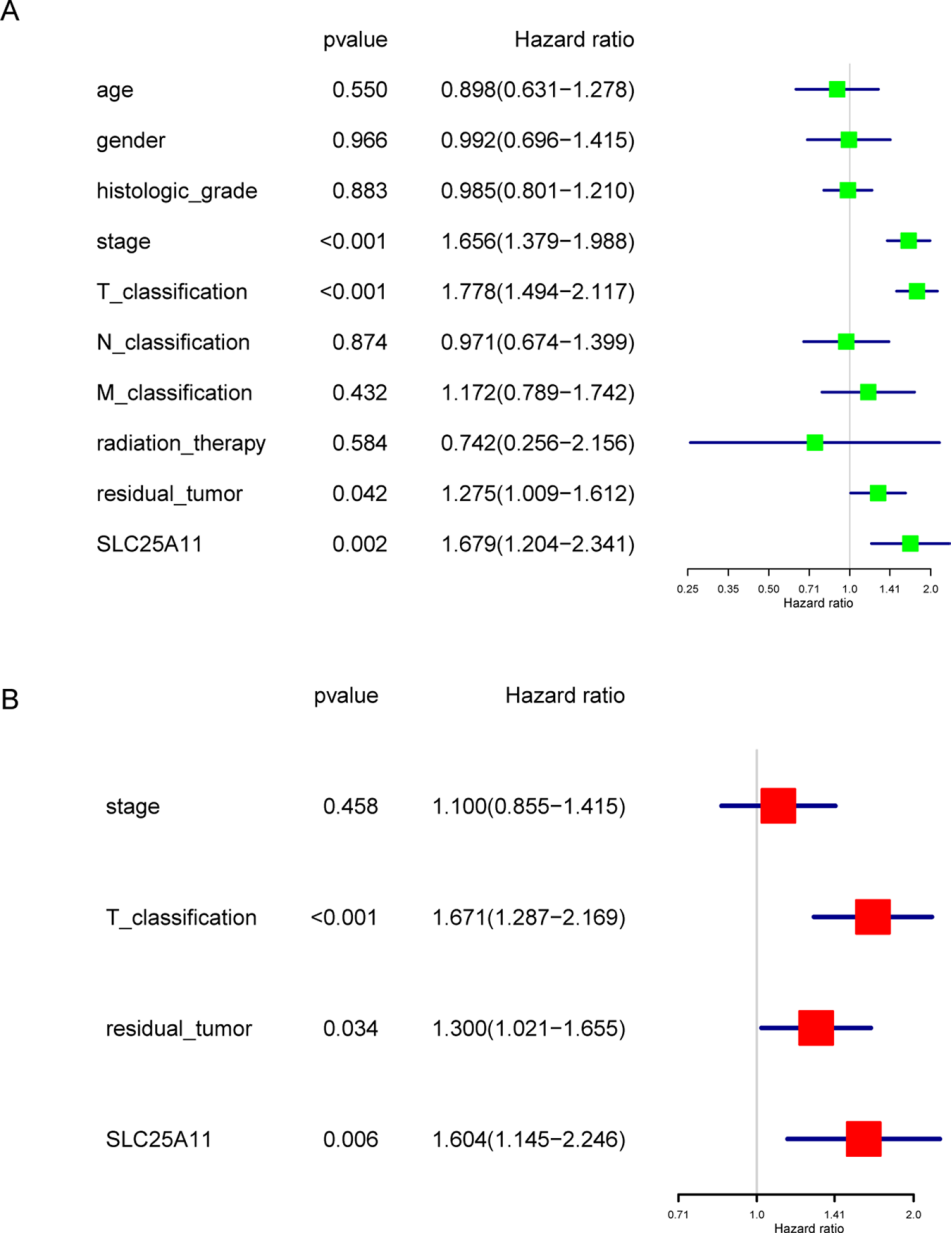
Cox model of RFS among liver cancer patients. Univariate and multivariate analysis of the correlation of SLC25A11 expression with RFS among liver cancer patients.

Univariate Cox analysis identified the potential RFS-related variables, including the stage, T classification, residual tumor status and SLC25A11. Multivariate Cox analyses showed that SLC25A11 expression (HR value: 1.604, 95% CI: 1.145–2.246, P = 0.006), residual tumor status (HR value: 1.300, 95% CI: 1.021–1.655, P = 0.034), and T classification (HR value: 1.671, 95% CI: 1.287–2.169, P < 0.001) were independent predictors of poor RFS (Fig. 7).

## Discussion

Among cancers worldwide, liver cancer has one of the worst prognoses. Many studies have investigated novel prognostic biomarkers in different types of cancer^10–26^. In this study, we demonstrated that SLC25A11 was low expressed in liver cancer by analyzing data from the TCGA. Low expression of SLC25A11 is related to poor prognosis. Both univariate and multivariate Cox analyses revealed that SLC25A11 played an important role in OS and RFS, which means that SLC25A11 is a novel independent prognostic factor in liver cancer.

In recent years, high SLC25A11 has been found in NSCLC and melanoma^8^. However, we found that SLC25A11 was expressed at low levels in liver cancer. ROC analysis suggested that SLC25A11 could be regarded as a marker in the diagnosis and prognosis of liver cancer. Moreover, we observed that histologic grade, clinical stage, and survival status were related to SLC25A11 expression, which confirmed the significance of this molecule.

SLC25A11, a member of the malate-aspartate shuttle (MAS), regulates the shuttling of 2-oxoglutarate by dicarboxylates from different tissues and cells using various methods^27–29^. These studies also provide evidence that SLC25A11 is a GSH carrier. In addition, SLC25A11 induction is a key strategy for maintaining mtGSH levels to limit ROS production in HCC^9^. Based on the findings that SLC25A11 expression is associated with clinical stage and histologic grade in liver cancer, we postulate that by stabilizing the mitochondrial membrane and maintaining sufficient mtGSH to withstand hypoxia-induced ROS production and cell apoptosis, SLC25A11 improves cell growth and tumor progression.

As one of the characteristics of cancer, fast growing cancer cells requires more ATP than normal cells. Because cancer cells rely mainly on glycolysis for energy, they maintain high mitochondrial oxidative phosphorylation for ATP generation and survival^30^. SLC25A11 has been proven to be a crucial transporter of NADH from the cytoplasm to mitochondria in the ATP production process, especially in cancer cells. Therefore, the association of the expression of SLC25A11 with poor survival may be due to the effect of SLC25A11 on tumor energy metabolism. This implies that SLC25A11 is necessary for embryogenesis but not for the proliferation of differentiated cells, which suggests that it can be a good marker for cancer.

To our knowledge, our research is the first to show the significant role of SLC25A11 in the prognosis of liver cancer by mining the TCGA database. We found that SLC25A11 could be regarded as a prognostic factor for poor survival in liver cancer. However, the total number of patients evaluated is a limitation of our research. There was no significant correlation between SLC25A11 expression and overall survival in ICGC and the GSE54236 database in Fig. 3. However, the statistical significance does not represent clinical significance because some variables could influence the P-value, such as sample size. For this study, we observed that low SLC25A11 expression was associated with poor prognosis in the ICGC and GSE54236 cohorts, but the P-value did not indicate significance, perhaps because of the sample size. We need to expand the sample size to confirm the prognostic value of SLC25A11 expression levels in the future. Furthermore, we postulate that SLC25A11 plays an essential role in transporting NADH and GSH from the cytoplasm into mitochondria, providing energy for liver cancer cells to withstand harsh environments. However, clinical trials and further experiments are needed to certify these results and clarify the specific molecular mechanism.

## Materials and Methods

### Data collection and mining

Clinical information and RNA-sequencing expression results were collected from the TCGA dataset, GEO dataset (https://www.ncbi.nlm.nih.gov/gds/), HCCDB dataset (http://lifeome.net/database/hccdb/) and ICGC dataset (https://icgc.org/). All these data were processed in R software (version 3.6.1) (https://www.r-project.org/) and related packages^31^.

### Statistical analysis

Box plots were used to evaluate the expression of SLC25A11 in the TCGA-Liver Hepatocellular Carcinoma (LIHC) dataset. A receiver operating characteristic curve (ROC) was drawn to assess the diagnostic significance of SLC25A11 expression by using the pROC package^32^. In accordance with the threshold identified by the ROC curve, liver cancer patients were separated into low and high expression groups. OS and RFS were compared between low and high groups via Kaplan-Meier analysis with the package in R^33,34^. Fisher’s exact and chi-square tests were utilized to evaluate correlations between the expression of SLC25A11 mRNA and clinical information. The potential prognostic factors were selected by Cox analysis. Correlations between SLC25A11 expression and survival and other clinical characteristics of patients were confirmed by using multifactor Cox analysis. *p < 0.05 was considered significant.

## Data Availability

All data generated or analyzed during this study are included in this published article.

## References

[1] Bray F (2018). Global cancer statistics 2018: GLOBOCAN estimates of incidence and mortality worldwide for 36 cancers in 185 countries. CA Cancer J. Clin..

[2] Simard EP, Ward EM, Siegel R, Jemal A (2012). Cancers with increasing incidence trends in the United States: 1999 through 2008. CA Cancer J. Clin..

[3] Gluer AM (2012). Systematic review of actual 10-year survival following resection for hepatocellular carcinoma. HPB (Oxford).

[4] Lash LH (2006). Mitochondrial glutathione transport: physiological, pathological and toxicological implications. Chem. Biol. Interact.

[5] Xu F, Putt DA, Matherly LH, Lash LH (2006). Modulation of expression of rat mitochondrial 2-oxoglutarate carrier in NRK-52E cells alters mitochondrial transport and accumulation of glutathione and susceptibility to chemically induced apoptosis. J. Pharmacol Exp. Ther.

[6] Rolo AP, Palmeira CM (2006). Diabetes and mitochondrial function: role of hyperglycemia and oxidative stress. Toxicol Appl Pharmacol.

[7] Wallace DC (2005). A mitochondrial paradigm of metabolic and degenerative diseases, aging, and cancer: a dawn for evolutionary medicine. Annu Rev Genet.

[8] Lee JS (2019). Loss of SLC25A11 causes suppression of NSCLC and melanoma tumor formation. EBioMedicine.

[9] Baulies A (2018). The 2-oxoglutarate carrier promotes liver cancer by sustaining mitochondrial GSH despite cholesterol loading. Redox Biol.

[10] Cai H (2019). Low CYP24A1 mRNA expression and its role in prognosis of breast cancer. Sci Rep.

[11] Cui Y, Jiao Y, Wang K, He M, Yang Z (2019). A new prognostic factor of breast cancer: High carboxyl ester lipase expression related to poor survival. Cancer Genet.

[12] Hou L (2019). ATP binding cassette subfamily B member 9 (ABCB9) is a prognostic indicator of overall survival in ovarian cancer. Medicine (Baltimore).

[13] Jiao Y, Fu Z, Li Y, Meng L, Liu Y (2018). High EIF2B5 mRNA expression and its prognostic significance in liver cancer: a study based on the TCGA and GEO database. Cancer management and research.

[14] Jiao Y, Fu Z, Li Y, Zhang W, Liu Y (2019). Aberrant FAM64A mRNA expression is an independent predictor of poor survival in pancreatic cancer. PloS one.

[15] Jiao Y (2019). OGDHL Expression as a Prognostic Biomarker for Liver Cancer Patients. Dis Markers.

[16] Jiao Y, Li Y, Ji B, Cai H, Liu Y (2019). Clinical Value of lncRNA LUCAT1 Expression in Liver Cancer and its Potential Pathways. J. Gastrointestin Liver Dis.

[17] Jiao Y, Li Y, Jiang P, Han W, Liu Y (2019). PGM5: a novel diagnostic and prognostic biomarker for liver cancer. PeerJ..

[18] Jiao Y, Li Y, Liu S, Chen Q, Liu Y (2019). ITGA3 serves as a diagnostic and prognostic biomarker for pancreatic cancer. OncoTargets and therapy.

[19] Jiao Y, Li Y, Lu Z, Liu Y (2019). High Trophinin-Associated Protein Expression Is an Independent Predictor of Poor Survival in Liver Cancer. Digestive diseases and sciences.

[20] Li Y (2019). High miR-454-3p expression predicts poor prognosis in hepatocellular carcinoma. Cancer management and research.

[21] Li Y, Jiao Y, Li Y, Liu Y (2019). Expression of La Ribonucleoprotein Domain Family Member 4B (LARP4B) in Liver Cancer and Their Clinical and Prognostic Significance. Dis Markers.

[22] Li Y, Jiao Y, Luo Z, Li Y, Liu Y (2019). High peroxidasin-like expression is a potential and independent prognostic biomarker in breast cancer. Medicine (Baltimore).

[23] Nie Y, Jiao Y, Li Y, Li W (2019). Investigation of the Clinical Significance and Prognostic Value of the lncRNA ACVR2B-As1 in Liver Cancer. BioMed Research International.

[24] Sun Z (2019). Low BCL7A expression predicts poor prognosis in ovarian cancer. J. Ovarian Res.

[25] Zhang, X., Cui, Y., He, M., Jiao, Y. & Yang, Z. Lipocalin-1 Expression as a Prognosticator Marker of Survival in Breast Cancer Patients. *Breast Care*, 10.1159/000503168 (2019). 10.1159/000503168 PMC7383281 32774222

[26] Zhao, Y. C. *et al*. Elevated high mobility group A2 expression in liver cancer predicts poor patient survival. *Rev Esp Enferm Dig* **112**, 10.17235/reed.2019.6365/2019 (2019). 10.17235/reed.2019.6365/2019 31823639

[27] Torres S (2017). Mitochondrial GSH replenishment as a potential therapeutic approach for Niemann Pick type C disease. Redox Biol.

[28] von Montfort C (2012). Mitochondrial GSH determines the toxic or therapeutic potential of superoxide scavenging in steatohepatitis. J. Hepatol.

[29] Wilkins HM, Brock S, Gray JJ, Linseman DA (2014). Stable over-expression of the 2-oxoglutarate carrier enhances neuronal cell resistance to oxidative stress via Bcl-2-dependent mitochondrial GSH transport. J. Neurochem.

[30] Alam MM, Lal S, FitzGerald KE, Zhang L (2016). A holistic view of cancer bioenergetics: mitochondrial function and respiration play fundamental roles in the development and progression of diverse tumors. Clin Transl Med.

[31] Samur MK (2014). RTCGAToolbox: a new tool for exporting TCGA Firehose data. PloS one.

[32] Robin X (2011). pROC: an open-source package for R and S+ to analyze and compare ROC curves. BMC Bioinformatics.

[33] Therneau, T. M. & April. A Package for Survival Analysis in S. (1994).

[34] Therneau, T. M. & Grambsch, P. M. Modeling Survival Data: Extending the Cox Model. Vol. 97 (Springer, 2000).

